# Radiography, CT, and MRI Diagnosis of Enzootic Nasal Tumor in Goats Infected With Enzootic Nasal Tumor Virus

**DOI:** 10.3389/fvets.2022.810977

**Published:** 2022-03-11

**Authors:** Ling-xu Li, Ying-jun Lv, Qing-yong Guo, Yun Liao, Yi-wen Guo, Ze-nan Su, Da-wei Yao, De-ji Yang

**Affiliations:** ^1^Diagnostic Imaging Center of Veterinary Medicine, College of Veterinary Medicine, Nanjing Agricultural University, Nanjing, China; ^2^College of Veterinary Medicine, Xinjiang Agricultural University, Urumqi, China

**Keywords:** enzootic nasal adenocarcinoma, enzootic nasal tumor virus, goat, magnetic resonance imaging, computed tomography

## Abstract

The aim of this study was to describe radiography, computed tomography (CT), and magnetic resonance imaging (MRI) findings of enzootic nasal tumors in goats infected with enzootic nasal tumor viruses. Five of six goats with a mean age of 2 years, showed clinical signs of respiratory disease. Head radiographs showed increased density of the unilateral or bilateral nasal cavity in four goats, and a CT scan showed that the space-occupying lesion of the nasal cavity originated from the ethmoid bone and was enhanced homogeneously postcontrast in all goats. The nasal concha was destroyed and the paranasal sinus mucosa was thickened and filled with fluid in some goats. On MRI, the mass exhibited equal or slightly higher signal intensity on T2 weighted images, equal signal intensity on T1 weighted images, a high signal on fluid-attenuated inversion recovery images and heterogeneous enhancement postcontrast. After dissection, histopathological examination of the mass and virus genome detection of the nasal secretions confirmed that the intranasal mass was a low-grade adenocarcinoma and that the goats were infected with enzootic nasal tumor virus type 2. In conclusion, CT and MRI have high diagnostic values for enzootic nasal tumors because they match the postmortem findings and are more accurate than radiography.

## Introduction

Enzootic nasal adenocarcinoma (ENA) is a neoplasia induced by viral infection in sheep and goats. It occurs naturally in many countries (1–3). Enzootic nasal tumor virus type 1 and enzootic nasal tumor virus type 2 (ENTV-2) are causative agents of ENA in sheep and goats, respectively, and both are β-retroviruses (4). ENTV-2 shares an oncogenic mechanism with enzootic nasal tumor virus type 1, and its oncogenic proprieties are associated with *env* gene products (5). These tumors have been classified histologically as low grade adenocarcinoma, and their target tissues are secretory epithelial and mesenchymal cells of the ethmoid bone in the nasal cavity (6). Electron microscopic and histochemical studies have shown that the tumors may originate either in olfactory or respiratory mucosal glands (7). Usually, the clinical symptoms are slowly progressive. Most of the nasal cavity is filled with tumor tissue before clinical symptoms appear. At necropsy, the tumors may be bilateral or more commonly unilateral. Gross evaluation, cytology, histopathology, PCR, and transmission electron microscopy are helpful for the diagnosis of ENA (8–10).

Imaging techniques are important diagnostic tools but have not always been used sufficiently in goats. Computed tomography (CT) scans can reveal the presence of a space-occupying lesion involving the nasal passage in small ruminants, and help in the diagnosis of ENA sheep lesions (3, 11, 12). It has been confirmed that magnetic resonance imaging (MRI) can depict soft tissue with high contrast in imaging examinations of the equine nasal cavity (13). MRI can show the size, extent and invasion of nasal soft tissue lesions clearly, and to some extent, allow for a judgement about the composition of the lesions (14). In this study, the radiographic, CT and MRI features of ENA were described in detail of six ENA goats, and the diagnostic value of imaging examination for ENA was discussed.

## Methods

### Animals

Except for one goat without obvious clinical symptoms, five goats began to show a mild runny nose for different durations (see Supplementary Table 1 for details). The next month, clinical symptoms developed, including snoring, sneezing, serious snot production, dyspnea, appetite loss and mental depression.

### Clinical and Laboratory Examinations

Routine clinical examination and hematological tests were performed immediately after admission to the hospital. Anticoagulated blood with ethylenediaminetetraacetic acid was used for complete blood counts, and blood serum was collected for biochemical examinations. Nasal secretions were sampled from the deep nasal cavity of all goats by using long sterile cotton swabs. The swab tips were preserved 4 mL of virus preservation solution (Hanks' balanced salt solution).

### Imaging Examinations

#### Anesthesia

Anesthesia (Jilin Huamu, Changchun, China) was induced with xylazine hydrochloride at 1 mL/kg by intravenous (IV) injection. Isoflurane (Ruiwode, Shenzhen, China) was used to maintain anesthesia. Rapid endotracheal intubation and the use of anesthesia machines were applied immediately after loss of consciousness. G6 died of respiratory arrest during the MRI scan without tracheal intubation.

#### Radiographs Examinations

After anesthesia, standard frontal and lateral radiographs of the head were taken with digital radiography system ARIA (Foschi, Italy) with 65 kV and 4.0 mAs parameters.

#### CT Examinations

The goats were scanned with the 16 slices Whole Body X-ray CT System Supria (Hitachi Medical Corporation, Tokyo, Japan) while lying prone under general anesthesia. The scan parameters were 120 kV, 300 mA, 0.6 mm slice thickness and 0.56 pitch, and the bone window (W/L: 2000/200) and soft tissue window (W/L: 400/50) of the head were reconstructed. Iohexol (350 mg/mL, Shukeming, Fuan Pharmaceutical, Chongqing, China) was administered as contrast medium by IV injection at a dosage of 1.85 mL/kg, and the injection speed was 3 mL/s. The arterial phase and delayed phase were scanned with the same scanning parameters.

#### MRI Examinations

The goats were scanned with the 1.5 T MRI system uMR 560 (United Imaging, Shanghai, China) while lying prone under general anesthesia. Spin echo T1 weighted images (SE T1WI), fast spin echo T2 weighted images (FSE T2WI) and fluid attenuated inversion recovery (FLAIR) images were obtained in the sagittal, transverse and coronal planes. The pulse sequence scanning conditions were as follows: SE T1WI (echo time (TE): 11 ms, repetition time (TR): 500 ms, number of signal averages (NSA): 1, echo train length: 1, slice thickness: 3 mm, acquisition matrix: 250 × 200); FSE T2WI (TE: 90 ms, TR: 7,000 ms, NSA: 1.6, echo train length: 13, slice thickness: 3 mm, acquisition matrix: 240 × 200); FLAIR (TE: 84 ms, TR: 7,600 ms, TI: 2,300 ms, NSA: 1, echo train length: 11, slice thickness: 3 mm, acquisition matrix: 220 × 170). Gadodiamide (0.5 mmol/mL, Omniscan, GE Healthcare, Ireland) was administered as contrast medium by manual IV injection at a dosage of 0.2 mL/kg. SE T1WI was scanned immediately after contrast injection.

### Dissection and Histopathological Examinations

After death, G1, G2, G3, G4, and G6 were subjected to head dissection. The goat head was cut along the paramedian sagittal plane, and the nasal septum was exposed and removed. Histopathological examination of samples from G1, G2, G3, and G6 were performed. The tissue samples were fixed in 10% neutral buffered formalin and embedded in paraffin wax using routine procedures. The dewaxed tissue sections were subjected to hematoxylin and eosin staining.

### Reverse Transcription PCR (RT-PCR) Detection of ENTV

Nasal secretion samples and blood samples were collected from the six affected goats and from two healthy goats at the time of admission and stored at −80°C for further use. Total RNA was extracted by RNAiso Plus (Takara, Dalian, China) and treated with an RT reagent Kit with gDNA Eraser (Takara, Dalian, China) to remove contaminating genomic DNA. The RNA was used to prepare cDNA as a template in RT-PCR. ENTV-2 gene-specific primers (F: 5′-TCCACCCTTCCTGGTGCC-3′ and R: 5′-CACAAACATGCCCTCGTCCCC-3′) amplified an 814 bp fragment consistent with published articles (15).

The PCR volume was 25 μL, containing 12.5 μL 2 × Es Taq MasterMix (CWBIO, Beijing, China), 1 μL each of the primers, 2.5 μL template and 8 μL double distilled water. The cycling conditions were as follows: 94°C for 5 min; 40 cycles of 94°C for 15 s, 65°C for 15 s and 72°C for 1 min; and a final extension of 5 min at 72°C. The products were examined by agarose gel electrophoresis.

## Results

### Clinical and Laboratory Examinations

Six goats exhibited clinical symptoms varying in severity (Supplementary Table 1). Upper respiratory symptoms of ENA goats are usually progressive. The goats showed unilateral or bilateral “runny nose” at the first sign. Over a period of time, the nasal fluid became viscous, and breath sounds coarsened, even worsening into dyspnea. The body temperature in all goats was normal and they were without any neurological symptoms. The blood cell count of G2 showed mild anemia (low hematocrit and low hemoglobin), and G3 suggested infection (high white blood cells, high neutrophil, and high lymphocyte). These abnormal routine blood tests may be related to a loss of appetite and secondary infection, respectively. Routine blood data of the other goats and the serum biochemistry data of all goats were within the normal range (Supplementary Table 2) (16).

### Imaging Diagnosis and Outcome

#### Radiographs Diagnosis

**Figure 1:**
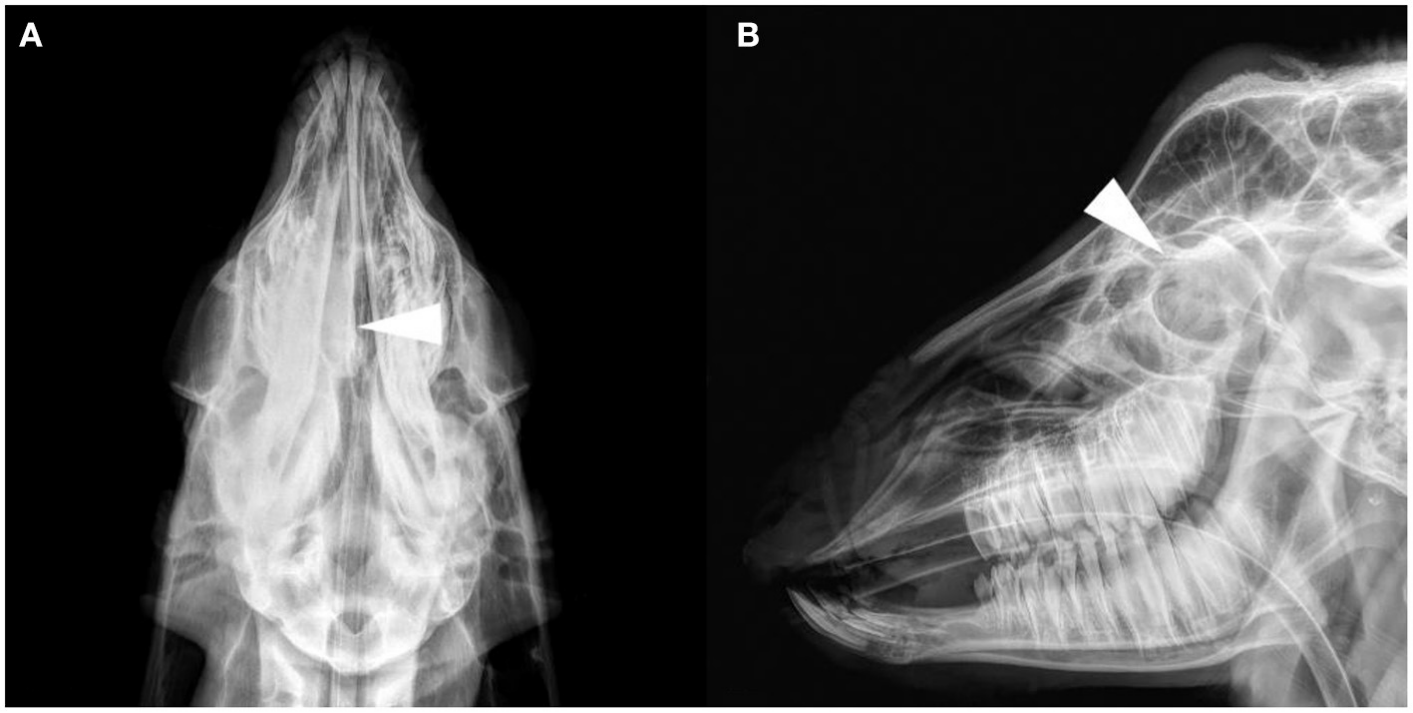
Radiograph images of the goat G2 head. **(A)** Dorsoventral projection. **(B)** Lateral projection. A soft tissue density image in the right nasal cavity, and the structure of the nasal concha was not clear (arrows).

The results of the frontal and lateral radiographic examination showed obvious lesions in the nasal cavity of four goats (see Supplementary Table 3 for details). Head anatomical structure name refer to published research (17, 18). Their characteristics were a soft tissue density mass from the middle to the caudal of the unilateral or bilateral nasal cavity, and the density increased in the nasal cavity (Figure 1). Frontal bone cortex discontinuity was only found in G6. No obvious changes in the nasal and frontal bones were observed in the other goats. Radiographic images cannot discriminate between tumors and infectious diseases.

#### CT Diagnosis

All goats underwent plain and contrast-enhanced head CT in our hospital. The major CT imaging findings are summarized in Supplementary Table 3. The CT scan showed a dense, soft tissue mass (~40 HU) with a space-occupying effect in the nasal cavity of all goats.

**Figure 2:**
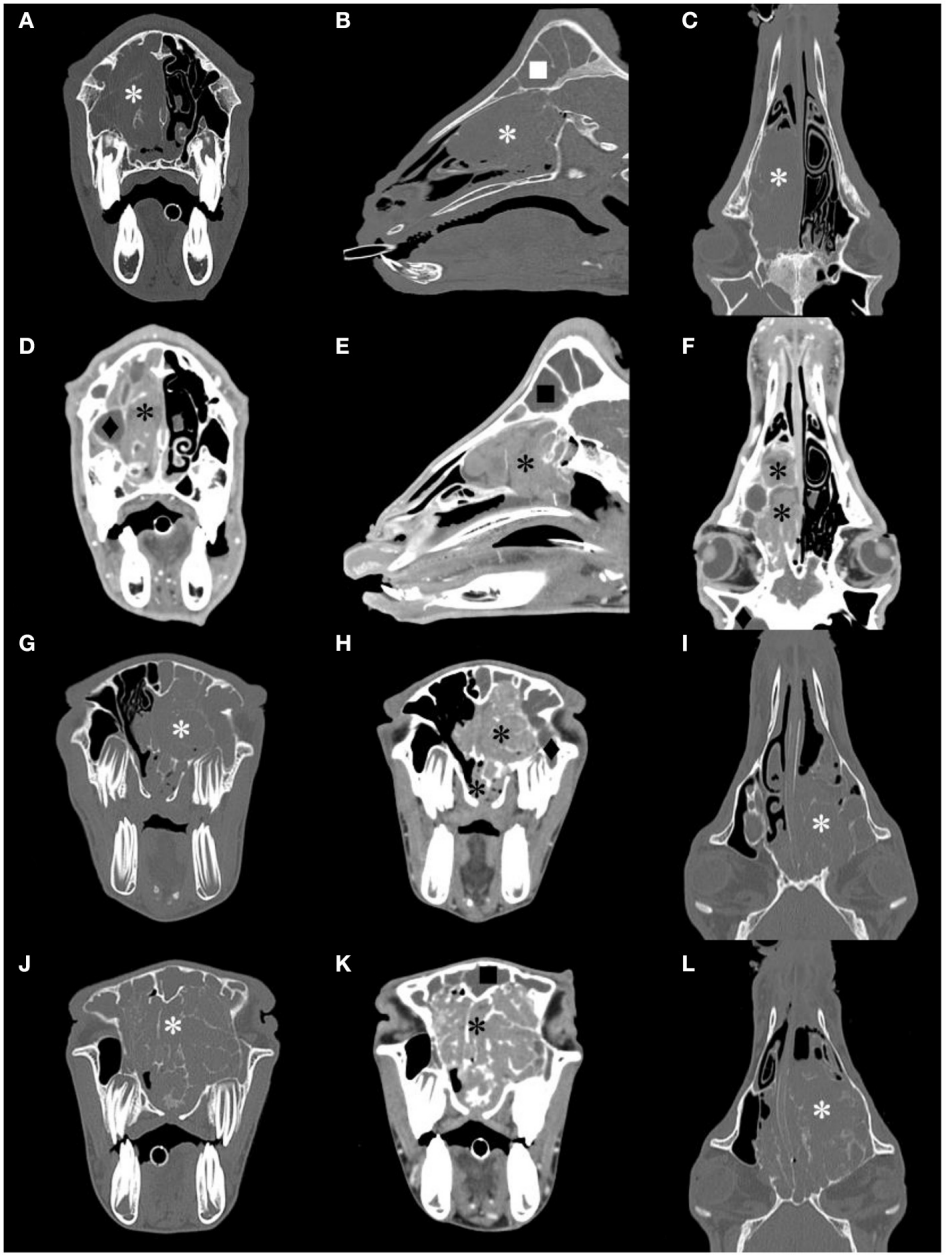
Computed tomography (CT) images of the goat G1, G2 and G3 head. Transverse, sagittal and dorsal bone window CT images [**(A–C)**, respectively], and the corresponding postcontrast-soft tissue window CT images [**(D–F)**, respectively] of G2. Transverse bone window and postcontrast-soft tissue window, and dorsal bone window CT images of G1 [**(G–I)**, respectively] and G3 [**(J–L)**, respectively]. A contrast-enhancement mass image in the nasal cavity, and the structure of the nasal concha and ethmoidal labyrinth were destroyed (*). The frontal sinus (■) and maxillary sinus(♦) were filled with fluid.

The lesion appeared lobulated, with clear boundaries, irregular edges, and uneven density. Unilateral cases often showed destruction and compression of the ethmoid and turbinate concha (Figures 2A–C). The mass in G5 was small, and it only invaded the ethmoid labyrinth. Cases where the mass invaded the contralateral side and bilateral cases showed obvious deviation or destruction of the nasal septum, and even invaded the nasopharynx to block the choanae (Figures 2G,I,J,L). Larger masses affected the other surrounding bony structures, such as the frontal bones and orbit.

After CT contrast, the mass was not evident in the arterial phase but showed moderate or intense (78–93 HU) continuous and homogeneous enhancement during the delayed phase (Figures 2D–F,H,K). In all goats, there was no non-enhanced necrotic area inside the mass. The fluid in the deformed nasal concha was contrasted clearly. The frontal sinus and maxillary sinus were filled with fluid with no contrast enhancement in five goats, and the mucosa of the paranasal sinus was thickened in three goats.

#### MRI Diagnosis

**Figure 3:**
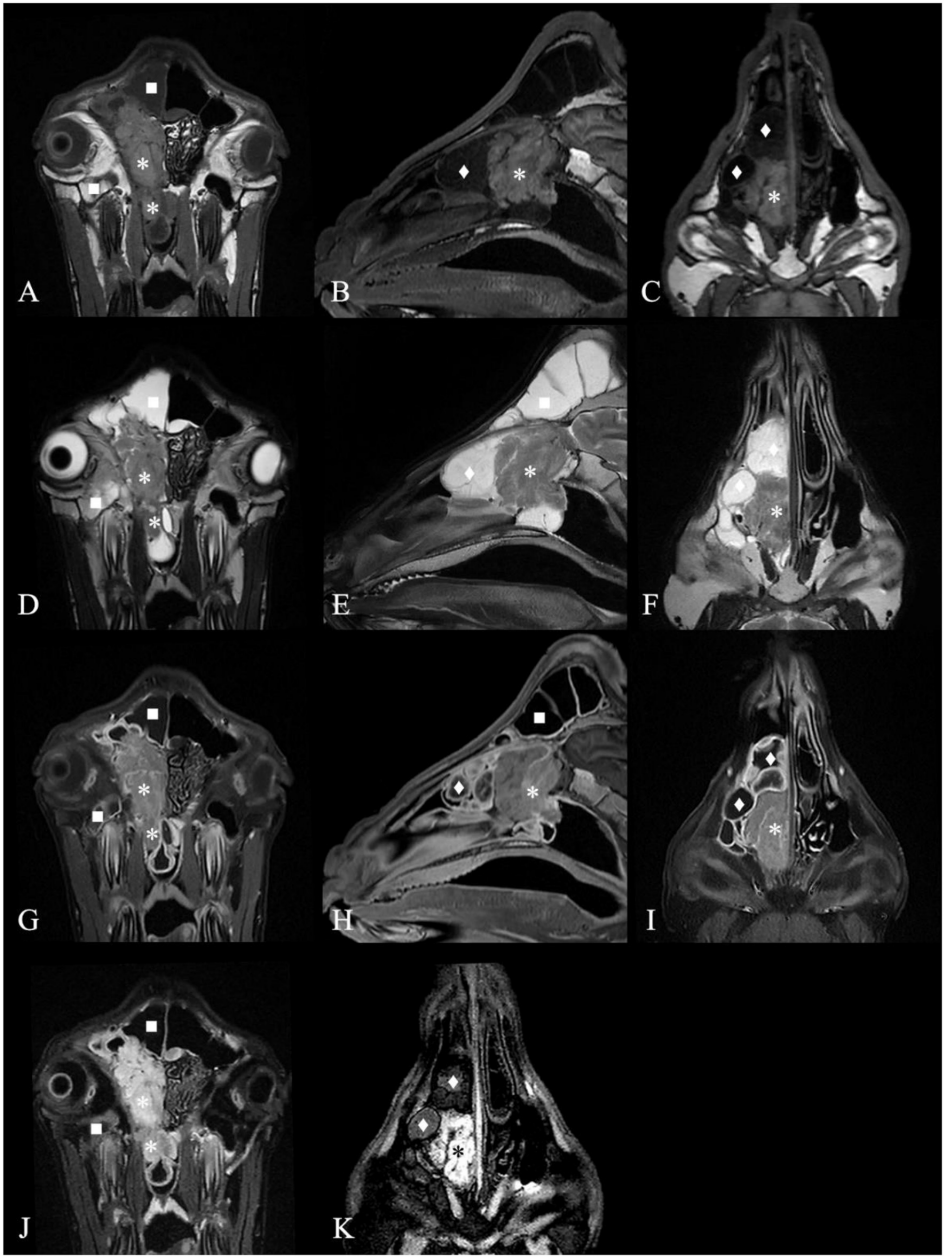
Magnetic resonance images of the goat G2 head. **(A)** T1 weighted image (T1WI), transverse plane. **(B)** T1WI, sagittal plane. **(C)** T1WI, coronal plane. **(D)** T2 weighted image (T2WI), transverse plan. **(E)** T2WI, sagittal plane. **(F)** T2WI, coronal plane. **(G)** Contrast-enhanced T1 weighted image (T1WI+C), transverse plane. **(H)** T1WI+C, sagittal plane. **(I)** T1WI+C, coronal plane. **(J)** Fluid attenuated inversion recovery (FLAIR) image, transverse plane. **(K)** FLAIR image, coronal plane. A contrast-enhancement mass with irregular boundary in the right nasal cavity, which showed equal signal intensity on T1WI and T2WI and high signal intensity on FLAIR (*) and made septum slightly shift to the left. The mass extended to the nasopharynx through the choana (*). The deformed nasal conchal bone was filled with fluid which showed low signal intensity on T1WI, high signal on T2WI and low signal on FLAIR (♦). The fluid in the right frontal sinus showed low signal intensity on T1WI, high signal on T2WI and low signal on FLAIR (■). Maxillary sinus hemorrhage showed low signal on T1WI, high signal on T2WI, and high signal on FLAIR (■).

Except for G6, which died during the MRI scan (G6 only plain scan), the other goats underwent plain and contrast-enhanced head MRI. Supplementary Table 3 summarizes the main MRI findings in the six ENA goats. MRI depicted the mass lesions more accurately. There was a mass with irregular boundaries in the unilateral or bilateral nasal cavity, which showed equal or slightly higher signal intensity on T2WI, an equal signal on T1WI, and a high signal intensity on FLAIR (Figure 3). The mass showed heterogeneous contrast enhancement on T1WI in all goats, and no significant area of non-enhancement was seen.

Most masses filled the ethmoidal labyrinth and squeezed the nasal concha, making the bilateral concha asymmetrical. The deformed nasal concha on the cranial and dorsolateral side of the mass was filled with mucin, which showed high signal intensity on T2WI, a low signal on T1WI, a slightly lower signal on FLAIR, and no contrast enhancement on T1WI in five goats. The signal intensity of the fluid in the paranasal sinuses was the same. The mucosa of the deformed nasal concha around the mass and mucosa of the affected frontal sinus were obviously homogeneously enhanced. This finding was suggestive of secondary sinusitis in these goats.

Compared with CT, the signal appearance of the mass on MRI was more similar among the six goats.

### Dissection and Histopathological Examinations

Dissection examination showed that the frontal sinus was filled with gelatinous material (Supplementary Figure 1). A large mass in the nasal cavity was closely connected with the cribriform plate and extended to the nasopharynx. The shape of the mass was irregular, white or pink, and without hemorrhage. The dorsal nasal concha was deformed and congested, and the same gel-like substance could be seen by incision into the dorsal nasal concha.

**Figure 4:**
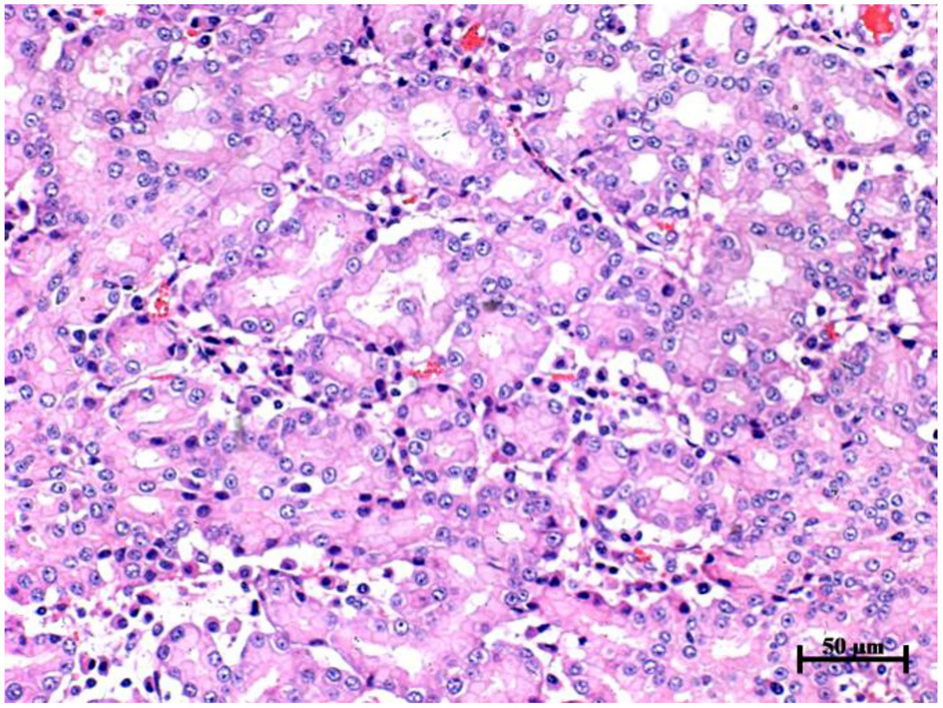
Histopathology of nasal mass in goat G2, hematoxylin and eosin stain, 20 × objective. The cells were predominantly arranged in a tubular fashion. The tubule was lined by simple columnar epithelium with distinct cell borders and a moderate amount of eosinophilic to amphophilic cytoplasm. The nuclei were round to oval and central to eccentrically located. Mitotic figures were rare, less than 1 per 10 high powered fields.

Histopathological examination showed that the surface of the nasal mass was covered with pseudostratified ciliated columnar epithelium (Figure 4). The nasal mass was comprised of loose connective tissue, capillary vessels, adenoid structures, lymphocytes and plasma cells. The cells were predominantly arranged in a tubular fashion. The tubules were lined by simple columnar epithelium with distinct cell borders and a moderate amount of eosinophilic to amphophilic cytoplasm. The nuclei were round to oval and central to eccentrically located. Mitotic figures were rare, <1 per 10 high-powered fields. Histopathology performed on the necropsy tissue samples confirmed the diagnosis of nasal adenocarcinoma.

### RT-PCR Detection of ENTV

ENTV-2 gene-specific primers amplified an 814 bp fragment of the same size from the nasal secretion samples of the six goats affected by ENA. RT-PCR results showed no ENTV-2 fragment in nasal secretion samples from two healthy goats and in all goat blood samples (Supplementary Figure 2). However, blood samples were collected only once at the time of admission, and it is not certain whether the virus exists in the blood of ENA goats at various periods after infection. Nasal secretion samples of six ENA goats were collected at various periods after admission. The PCR results of the nasal secretion sample collected from G5 at the first time were negative (data not shown). The nasal secretion samples of the other goats at different periods were randomly examined by RT-PCR, and the results were all positive (data not shown). Furthermore, total RNA was extracted and subjected to direct reverse transcription, and all samples from all goats (including blood samples and nasal secretion samples from ENA-affected goats and healthy goats) were PCR positive. Therefore, it is important during RT-PCR detection of ENTV to remove all potential DNA contamination.

## Discussion

ENA is a progressive nasal tumor in goats and sheep. Current studies have shown that nasal adenocarcinoma originates from the ethmoid mucosa in goats and sheep and is very similar to most nasal adenocarcinomas in humans and small animals (19, 20). There are no effective prevention and treatment methods for ENA, so early diagnosis is particularly important. In this study, the imaging characteristics of radiography, plain and contrast-enhanced CT and MRI in six goats with ENA were described.

In the present study, nasal lesions were found in only four of six ENA goats on radiographic images. On radiography, the nasal cavity lesions on the dorsoventral plane were more obvious than those on the lateral plane. Due to the complex structures and overlapping tissues of the nasal cavity, the masses in unilateral cases are not obvious on the lateral plane, and when the mass is small, radiographic examination cannot diagnose the space-occupying lesion of the nasal cavity. Another limitation of radiography is that it is impossible to judge whether there are space-occupying lesions in the nasopharynx due to occlusion of the dente molares on the lateral plane. Therefore, it is better to diagnose ENA with frontal radiography than lateral radiography.

In this study, the tumors could be diagnosed accurately by CT and MRI in all six goats, even early lesions of the nasal cavity without clinical symptoms. According to the imaging examination results, the nasal cavity lesions of six goats showed the following three types: unilateral and only located in the ethmoidal labyrinth (G5), unilateral and nasal concha destruction (G2, G4, and G5), and bilateral and severe ethmoid bone and nasal concha destruction (G1, G3, and G6).

On plain CT scan, the ethmoid bone and nasal concha in the nasal cavity show destruction and/or compression according to the size of the mass. Pathological changes in other bony structures are more common in severe cases. Orbit compression and frontal bone deformation can be found in this study. Contrast-enhanced CT can help to distinguish mass margins from the sinus fluid and normal adjacent soft tissues. After contrast enhancement, the mass is intensely enhanced during the delayed phase with clear boundaries. The method of using CT contrast agent in this study was based on the experience of small animal cases in our hospital. Compared with the dose used in previous research (21), we used less contrast agent and achieved a good effect.

On MRI, all goats had similar signal intensities, the signal performance of the tumor was equal to or high on T2WI, equal on T1WI, high on FLAIR, and heterogeneously enhanced on T1WI, consistent with the criteria for adenocarcinoma (22). On CT and MRI, no areas of unenhanced necrosis or significantly enhanced areas were seen in the six goats, which is different from the images of high-grade nasal tumors in dogs, cats and horses (20, 22, 23). There are few studies on goat MRI contrast enhancement, and in goats, it is mostly used to diagnose brain diseases (24). Nasal adenocarcinoma in dogs and cats is usually examined after IV administration of MRI contrast agent to assess whether there is potential intra- and extracranial extension (25). This study and previous ENA goat necropsy revealed that ENA hardly invaded the cranial cavity in goats and sheep (12). In this research, the effect of the MRI contrast agent on enhancing tumors was limited. The location, size, shape, and boundary of the tumor can already be well evaluated with conventional T2WI, T1WI, and FLAIR sequences (Figure 3). However, contrast-enhanced MRI has good diagnostic value for secondary sinusitis caused by the mass in ENA goats. To the best of our knowledge, this is the first study to use MRI for the evaluation of ENA goats. According to the results of the imaging examination, ENA is a malignant tumor that invades the nasal cavity of goats and causes the destruction of the nasal concha bone and ethmoidal labyrinth, blockage of the nasal meatus and choana, and secondary sinusitis.

Other common goat respiratory diseases include contagious caprine pleuropneumonia, goat Pasteurellosis, goat parainfluenza virus type 3, chronic proliferative rhinitis, etc. Naturally occurring ENA has clinical symptoms similar to other respiratory diseases, such as runny nose, snoring and sneezing. The main lesion of the nasal cavity in these diseases may look like a space-occupying lesion. In chronic proliferative rhinitis sheep, swollen ventral, dorsal and ethmoidal nasal concha can be observed with CT (26), but the lesions are mostly located in the ventral nasal concha and are rare in the ethmoid bone unlike ENA (13).

It should be noted that G5 did not exhibit obvious clinical symptoms, and its first PCR result was negative (data not shown), but CT and MRI confirmed ENA. By repeating the PCR detection, the result was eventually positive. Therefore, CT and MRI have high clinical value for the early diagnosis of ENA.

In conclusion, plain CT and MRI scans can accurately diagnose ENA, and contrast examination can help further assess tumors and secondary sinusitis. CT and MRI can clearly show the location, size, boundary and invasion of nasal tumors in ENA goats. Considering the cost of contrast agents, and the longer duration of MRI increasing the risk of death from anesthesia, plain CT scans should be regarded as the gold standard for the diagnosis of ENA in goats. This is the first study to diagnose ENA with ENTV-2 infection by radiographs, CT and MRI in goats.

## Data Availability Statement

The datasets presented in this study can be found in online repositories. The names of the repository/repositories and accession number(s) can be found in the article/Supplementary Material.

## Ethics Statement

The animal study was reviewed and approved by Committee on the Ethics of Animal Experiments of Nanjing Agricultural University. Written informed consent was obtained from the owners for the participation of their animals in this study.

## Author Contributions

L-xL, Y-jL, Q-yG, YL, Y-wG, and Z-nS contributed to the acquisition of data, data analysis, and drafting of the manuscript. D-wY and D-jY contributed to the concept and design, data analysis and interpretation, and critical revision of the manuscript. All authors contributed to the article and approved the submitted version.

## Funding

This work was supported by the Fundamental Research Funds for the Central Universities (KYYJ202104).

## Conflict of Interest

The authors declare that the research was conducted in the absence of any commercial or financial relationships that could be construed as a potential conflict of interest.

## Publisher's Note

All claims expressed in this article are solely those of the authors and do not necessarily represent those of their affiliated organizations, or those of the publisher, the editors and the reviewers. Any product that may be evaluated in this article, or claim that may be made by its manufacturer, is not guaranteed or endorsed by the publisher.

## Supplementary Material

Supplementary Figure 1 Dissection pictures of the goat G2 head. **(A)** Cutting from the paramedian sagittal plane. **(B)** Removing the nasal septum and exposing mass. **(C)** Removing the mass and cutting the nasal concha bone. An irregular, white or pink, and without hemorrhage mass in the right nasal cavity, which was closely connected with the cribriform plate and extended to the nasopharynx. The right dorsal nasal concha was deformed and congested, and the gel-like substance can be seen in the dorsal nasal concha.

Supplementary Figure 2 Reverse transcription PCR identification showed that enzootic nasal tumor virus type 2 (ENTV-2) fragment (814 bp) were amplified from nasal secretion samples in six affected goats, but no ENTV-2 fragment in two healthy goats. No ENTV-2 fragment was amplified from blood samples in all goats. Lanes 1, 3, 5, 7, 9, 11, 13, and 15, nasal secretion samples from G1-G6 and two healthy goats; lanes 2, 4, 6, 8, 10, 12, 14, and 16, blood samples from G1-G6 and two healthy goats; lanes 17, blank control; lanes M, DNA marker (2000, 1000, 750, 500, 250, 100 bp).

Supplementary Table 1 Basic clinical information of six goats.

Supplementary Table 2 Blood routine and blood serum biochemistry results of six goats.

Supplementary Table 3 Radiography, computed tomography (CT) and magnetic resonance imaging (MRI) features of six goats.
