# Supplementary figures and images for: Radiography, CT, and MRI Diagnosis of Enzootic Nasal Tumor in Goats Infected With Enzootic Nasal Tumor Virus
Source: Front Vet Sci. 2022 Mar 11;9:810977. doi: 10.3389/fvets.2022.810977 (PMC8963243; doi:10.3389/fvets.2022.810977)

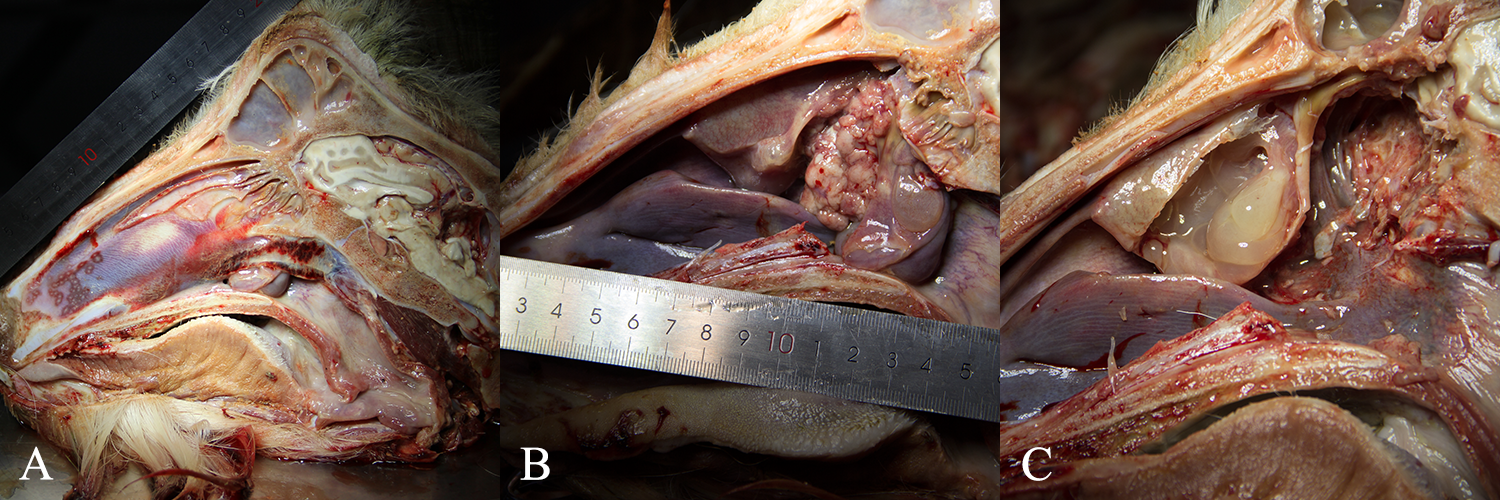

Supplement: Supplementary Figure 1 — Dissection pictures of the goat G2 head. (A) Cutting from the paramedian sagittal plane. (B) Removing the nasal septum and exposing mass. (C) Removing the mass and cutting the nasal concha bone. An irregular, white or pink, and without hemorrhage mass in the right nasal cavity, which was closely connected with the cribriform plate and extended to the nasopharynx. The right dorsal nasal concha was deformed and congested, and the gel-like substance can be seen in the dorsal nasal concha. [file Image_1.TIF]

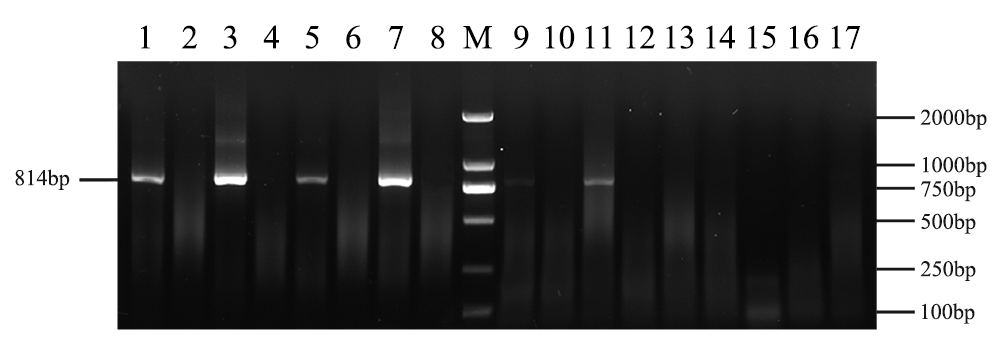

Supplement: Supplementary Figure 2 — Reverse transcription PCR identification showed that enzootic nasal tumor virus type 2 (ENTV-2) fragment (814 bp) were amplified from nasal secretion samples in six affected goats, but no ENTV-2 fragment in two healthy goats. No ENTV-2 fragment was amplified from blood samples in all goats. Lanes 1, 3, 5, 7, 9, 11, 13, and 15, nasal secretion samples from G1-G6 and two healthy goats; lanes 2, 4, 6, 8, 10, 12, 14, and 16, blood samples from G1-G6 and two healthy goats; lanes 17, blank control; lanes M, DNA marker (2000, 1000, 750, 500, 250, 100 bp). [file Image_2.TIF]
