# Supplementary material for: Radiography, CT, and MRI Diagnosis of Enzootic Nasal Tumor in Goats Infected With Enzootic Nasal Tumor Virus
Source: Front Vet Sci. 2022 Mar 11;9:810977. doi: 10.3389/fvets.2022.810977 (PMC8963243; doi:10.3389/fvets.2022.810977)
Supplement: Supplementary Table 1 — Basic clinical information of six goats. [file Table_1.DOCX]

**Supplementary Table 1** **Basic clinical information of six goats.**

| **Goat number** | **G1** | **G2** | **G3** | **G4** | **G5** | **G6** |
| --- | --- | --- | --- | --- | --- | --- |
| **Age** | 2.0 years | 3.0 years | 2.0 years | 1.0 years | 1.5 years | 2.0 years |
| **Sex** | Female | Male | Female | Female | Female | Male |
| **Weight** | 23.0 kg | 47.3 kg | 20.1 kg | 18.9 kg | 17.6 kg | 29.5 kg |
| **Breed** | Anhui white goat | Anhui white goat | Anhui white goat | Anhui white goat | Anhui white goat | Anhui white goat |
| **Time of first**  **clinical signs** | Unknown | Three months ago | A month ago | About a month ago | No obvious clinical signs | Three months ago |
| **Clinical signs**  **at admission** | Serous nasal fluids bilateral,  heavy breath sounds,  foam around the mouth,  mental depression | Serous nasal fluids with right nostril,  heavy breath sounds,  slight loss of appetite | Serous nasal fluids bilateral,  heavy breath sounds,  salivation,  severe mental depression,  moderate loss of appetite | A little transparent liquid around the right nostril | No obvious clinical signs | Mucoserous nasal fluids bilateral,  facial swelling,  open mouth breathing,  severe salivation and mental depression,  no appetite |
| **Treatment**  **and**  **effect** | Conventional antibiotic treatment was ineffectual | No treatment | Tracheotomy for relieve dyspnea | No treatment | No treatment | Conventional antibiotic treatment was ineffectual |
| **Outcome** | Died of respiratory failure 28 days after admission | Died of respiratory failure 41 days after admission | Died of malnutrition 16 days on postoperative | Died of suffocation 53 days after admission | Not dead | Died of respiratory arrest during MRI scan after admission |
