# Supplementary material for: Radiography, CT, and MRI Diagnosis of Enzootic Nasal Tumor in Goats Infected With Enzootic Nasal Tumor Virus
Source: Front Vet Sci. 2022 Mar 11;9:810977. doi: 10.3389/fvets.2022.810977 (PMC8963243; doi:10.3389/fvets.2022.810977)
Supplement: Supplementary Table 2 — Blood routine and blood serum biochemistry results of six goats. [file Table_2.DOCX]

**Supplementary Table 2 Blood routine and blood serum biochemistry results of six goats**

| **Parameters** | **Normal range** (16) | **Goat number and results** | | | | | |
| --- | --- | --- | --- | --- | --- | --- | --- |
|  |  | **G1** | **G2** | **G3** | **G4** | **G5** | **G6** |
| **White blood cell count** (10^9/L) | 11.32-18.54 | 11.32 | 13.07 | 23.93↑ | 17.97 | 12.20 | 12.74 |
| **Neutrophil count** (10^9/L) | 1.20-7.20 | 4.88 | 5.83 | 13.82↑ | 4.35 | 4.33 | 6.36 |
| **Lymphocyte count** (10^9/L) | 2.00-9.00 | 4.91 | 2.60 | 9.18↑ | 3.07 | 4.27 | 5.15 |
| **Monocyte count** (10^9/L) | 0-0.55 | 0.26 | 0.30 | 0.55 | 0.22 | 0.54 | 0.09 |
| **Eosinophil count** (10^9/L) | 0.05-0.65 | 0.24 | 0.33 | 0.10 | 0.32 | 0.23 | 0.12 |
| **Basophil count** (10^9/L) | 0-0.12 | 0.03 | 0.01 | 0.11 | 0.01 | 0.01 | 0.02 |
| **Red blood cell count** (10^12/L) | 8.30-17.90 | 17.38 | 11.44 | 14.51 | 12.98 | 14.91 | 12.90 |
| **Hemoglobin** (g/L) | 76.50-103.71 | 102.00 | 63.00↓ | 85.00 | 84.00 | 92.00 | 79.00 |
| **H****ematocrit** (%) | 23.00-35.00 | 28.70 | 17.10↓ | 23.60 | 23.40 | 25.30 | 23.10 |
| **Mean corpuscular volume** (fL) | 14.00-25.00 | 16.50 | 14.90 | 16.20 | 18.00 | 17.00 | 17.90 |
| **Mean corpuscular hemoglobin** (pg) | 5.20-8.00 | 5.90 | 5.50 | 5.90 | 6.50 | 6.10 | 6.10 |
| **Glucose** (mmol/L) | 2.77-4.44 | 3.83 | 4.02 | 4.20 | 4.11 | 3.39 | 4.39 |
| **Albumin** (g/L) | 27.00-39.00 | 35.10 | 38.50 | 30.40 | 34.40 | 31.30 | 38.10 |
| **Alkaline phosphatase** (U/L) | 75.45-313.19 | 78.90 | 89.80 | 107.20 | 93.90 | 80.30 | 132.50 |
| **Alanine aminotransferase** (U/L) | 24.00-38.00 | 35.40 | 28.80 | 25.40 | 24.50 | 25.60 | 27.20 |
| **Aspartate aminotransferase** (U/L) | 99.89-136.92 | 103.30 | 109.10 | 107.20 | 118.30 | 105.90 | 129.50 |
| **Creatine kinase** (U/L) | 194.96-374.08 | 302.10 | 248.00 | 289.00 | 199.30 | 232.00 | 270.00 |
| **Creatinine** (μmol/L) | 36.91-61.14 | 46.90 | 55.50 | 50.20 | 40.90 | 41.70 | 42.30 |
| **Serum calcium** (mmol/L) | 2.10-3.10 | 2.66 | 2.34 | 2.75 | 2.53 | 2.31 | 2.93 |
| **Gamma-glutamyltransferase** (U/L) | 54.13-71.59 | 59.20 | 55.50 | 60.20 | 67.10 | 55.10 | 60.50 |
| **Serum magnesium** (mmol/L) | 0.31-1.48 | 1.11 | 1.08 | 1.03 | 1.10 | 1.04 | 1.12 |
| **Total protein** (g/L) | 70.00-86.12 | 72.60 | 84.80 | 77.50 | 80.60 | 78.70 | 84.20 |

↓, lower than normal range; ↑, higher than normal range.
