# Supplementary material for: Radiography, CT, and MRI Diagnosis of Enzootic Nasal Tumor in Goats Infected With Enzootic Nasal Tumor Virus
Source: Front Vet Sci. 2022 Mar 11;9:810977. doi: 10.3389/fvets.2022.810977 (PMC8963243; doi:10.3389/fvets.2022.810977)
Supplement: Supplementary Table 3 — Radiography, computed tomography (CT) and magnetic resonance imaging (MRI) features of six goats. [file Table_3.DOCX]

**Supplementary Table 3 Radiography, computed tomography (CT) and magnetic resonance imaging (MRI) features of six goats.**

| **Images features** | **Goat number** |
| --- | --- |
| **Radiography images features** | |
| Increased nasal cavity density | G1 (left), G2 (right), G3 (bilateral) and G6 (bilateral) |
| Discontinuous bone cortex of frontal bone | G6 |
| Shifted or blurred nasal septum | G1 (slightly shifted), G3 (blurred) and G6 (blurred) |
| **Plain CT images features** | |
| Mass location |  |
| Bilateral | G1 (most left), G3 and G6 |
| Unilateral | G2 (right), G4 (right) and G5 (right) |
| Lesion invasion |  |
| Nasopharynx | G1, G2, G3, and G6 |
| Ethmoid bone | G1, G2, G3, G4, G5 (only ethmoidal labyrinth) and G6 |
| Nasal septum | G1, G2, G3 and G6 |
| Nasal concha | G1, G2, G3, G4 and G6 |
| Other bony structures | G1 (orbit), G3 (orbit and frontal bone) and G6 (orbit and frontal bone) |
| Paranasal sinuses |  |
| Frontal sinus transudate | G1, G2, G3, G4 (little) and G6 (also compression) |
| Ethmoidal sinus destruction | G1, G2, G3, G4 (little) and G6 |
| Maxillary and palatine sinus transudate | G1, G2, G3 (also compression) and G6 |
| **Contrast-enhanced CT images features** | |
| Enhancement of mass during delayed phase | G1 (intense), G2 (intense), G3 (moderate), G4 (moderate), G5 (moderate) and G6 (intense) |
| Mild enhancement edema around the mass | G1, G2, G3, G4 and G6 |
| Paranasal sinuses with mucosal thickening | G2 (frontal sinus), G4 (frontal sinus) and G6 (frontal and maxillary sinus) |
| **Plain and contrast-enhanced MRI images features*** | |
| Signal characteristics of mass | |
| Equal or slightly higher signal intensity on T2WI, equal signal on T1WI and high signal on FLAIR | G1, G2, G3, G4, G5 and G6 |
| Heterogeneous enhancement | G1, G2, G3, G4 and G5 |
| Signal characteristics of mucin around the mass and in paranasal sinuses | |
| High signal intensity on T2WI, low signal on T1WI and slightly lower signal on FLAIR | G1, G2, G3 and G6 |
| No enhancement | G1, G2 and G3 |
| Signal characteristics of mucosal of frontal sinus | |
| High signal intensity on T2WI, equal signal on T1WI and high signal on FLAIR | G2, G4 and G6 |
| Homogeneous enhancement | G2, G3 and G4 |

Head anatomical structure name and comparison refer to published research (17, 18).

* Contrast-enhanced MRI was performed in five goats, because G6 died during the plain MRI scan due to respiratory arrest.

T2WI, T2 weighted images; T1WI, T1 weighted images; FLAIR, fluid attenuated inversion recovery.
